# The prognostic value of health-related quality-of-life data in predicting survival in glioblastoma cancer patients: results from an international randomised phase III EORTC Brain Tumour and Radiation Oncology Groups, and NCIC Clinical Trials Group study

**DOI:** 10.1038/sj.bjc.6603876

**Published:** 2007-07-03

**Authors:** M Mauer, R Stupp, M J B Taphoorn, C Coens, D Osoba, C Marosi, R Wong, O de Witte, J G Cairncross, F Efficace, R O Mirimanoff, P Forsyth, M J van den Bent, M Weller, A Bottomley

**Affiliations:** 1 European Organisation for Research and Treatment of Cancer, Quality of Life Unit, Data Center, Avenue Mounier 83/11, 1200 Brussels, Belgium; 2 Centre Hospitalier Universitaire Vaudois and University of Lausanne, rue du Bugnon 46, 1011 Lausanne, Switzerland; 3 Medical Center Haaglanden/Westeinde Hospital, Lijnbaan 32 Box 432, 2501 The Hague, The Netherlands; 4 QOL consulting, Edendale Court 4939, West Vancouver V7W 3H7, Canada; 5 Medical University of Vienna, Waehringer Guertel 18-20, 1090 Vienna, Austria; 6 Hamilton Health Sciences, Juravinski Cancer Center, Concession Street 699, Hamilton, Ontario L8V 5C2, Canada; 7 Hopital Universitaire Erasme, Route de Lennik 808, 1070 Brussels, Belgium; 8 Department of Clinical Neurosciences, University of Calgary and Foothills Hospital, Alberta, Canada; 9 Tom Baker Cancer Center, University of Calgary, 1331 29th Street N.W., Calgary, AB T2N 4N2, Canada; 10 Erasmus University Medical Center, Postbus 5201, 3008 AE Rotterdam, The Netherlands; 11 Department of Neurology, University of Tübingen, Hoppe-Seyler-Str. 3, 72076 Tübingen, Germany

**Keywords:** HRQOL scores, prognostic factors, glioblastoma

## Abstract

This is one of the few studies that have explored the value of baseline symptoms and health-related quality of life (HRQOL) in predicting survival in brain cancer patients. Baseline HRQOL scores (from the EORTC QLQ-C30 and the Brain Cancer Module (BN 20)) were examined in 490 newly diagnosed glioblastoma cancer patients for the relationship with overall survival by using Cox proportional hazards regression models. Refined techniques as the bootstrap re-sampling procedure and the computation of *C*-indexes and *R*^2^-coefficients were used to try and validate the model. Classical analysis controlled for major clinical prognostic factors selected cognitive functioning (*P*=0.0001), global health status (*P*=0.0055) and social functioning (*P*<0.0001) as statistically significant prognostic factors of survival. However, several issues question the validity of these findings. *C*-indexes and *R*^2^-coefficients, which are measures of the predictive ability of the models, did not exhibit major improvements when adding selected or all HRQOL scores to clinical factors. While classical techniques lead to positive results, more refined analyses suggest that baseline HRQOL scores add relatively little to clinical factors to predict survival. These results may have implications for future use of HRQOL as a prognostic factor in cancer patients.

It has become increasingly accepted that, in addition to the traditional assessment of clinical outcomes, health-related quality-of-life (HRQOL) information can play a key role in cancer research and may help in individual patient care (Detmar *et al*, 2002; Velikova *et al*, 2004). Patients' self-assessment of HRQOL is now an established end point for treatment comparisons in many cancer disease sites (Bottomley *et al*, 2003), particularly in advanced disease (Efficace *et al*, 2004a; Bottomley *et al*, 2005).

Theoretically, HRQOL outcomes could have various clinical applications, including supporting clinical decision-making by providing the patient's perspective or providing prognostic information. Recent studies have shown that HRQOL parameters can be independent prognostic factors for survival in cancer patients (Kramer *et al*, 2000). If HRQOL parameters are independent predictors of survival, they could be used in daily practice to identify patients who will benefit from a specific intervention; further, it could help to avoid over-treatment of patients who will gain no benefit from, often, toxic and aggressive therapies or to set up more tailored psychosocial intervention programmes aimed at improving patients' HRQOL. Furthermore, they could be used to better stratify patients in future cancer clinical trials, hence better interpreting study outcomes or helping identify critical areas that could help in the selection of key end points for future clinical trials.

Health-related quality-of-life prognostic factor analyses have been carried out on several different cancer populations, including, among others, lung (Herndon *et al*, 1999; Langendijk *et al*, 2000; Montazeri *et al*, 2001; Efficace *et al*, 2006), oesophageal (Blazeby *et al*, 2001; Fang *et al*, 2004), advanced breast (Luoma *et al*, 2003; Efficace *et al*, 2004a), and head and neck (de Graeff *et al*, 2001) cancers, highlighting the importance HRQOL scores may have in predicting survival. Only three studies have examined HRQOL and/or cognitive functioning as a prognostic factor in brain cancer (Meyers *et al*, 2000; Klein *et al*, 2003; Sehlen *et al*, 2003). The present study evaluates the prognostic value of HRQOL data collected from a prospective, large-scale international randomised controlled trial, by using various statistical techniques in an attempt to provide robust conclusions on the prognostic value of HRQOL in glioblastoma multiforme patients.

## METHODS

### Treatment

In this international, multicentre study (EORTC trial 26981-22981-NCIC CE3), 573 patients with newly diagnosed glioblastoma were randomised to treatment with radiotherapy (RT) only or RT with concomitant temozolomide (TMZ) chemotherapy followed by six cycles of TMZ chemotherapy. Patients were stratified for institution, performance status (WHO performance status 0 or 1 *vs* 2), age (<50 *vs* ⩾50 years) and the extent of the resection at surgery (biopsy only *vs* debulking surgery/resection). The details on trial conduct and clinical outcome have previously been reported (Hegi *et al*, 2005; Stupp *et al*, 2005; Mirimanoff *et al*, 2006). The trial was approved by the EORTC protocol review committee and the ethics committee of each participating centre. All patients provided written informed consent.

### Procedures

#### Health-related quality-of-life evaluations

The primary end point of the clinical trial was survival, with HRQOL being a secondary end point. Two HRQOL measures were selected for the trial: the EORTC Quality of Life Questionnaire C30 (EORTC QLQ-C30, version 2) (Aaronson *et al*, 1993) and the EORTC QLQ-Brain Cancer Module (QLQ-BN20) (Osoba *et al*, 1996). Both tools have robust psychometric properties resulting from rigorous testing and development from their use in several international cancer clinical trials (Bottomley *et al*, 2003). The EORTC QLQ-C30 is a core measure designed to be supplemented with disease-specific questionnaires. The EORTC QLQ-BN20 was developed specifically in patients with brain cancer. Both instruments were available in the language of all participating patients (Cull *et al*, 2002).

The EORTC QLQ-C30 measure comprises five functioning scales – physical, role, emotional, cognitive and social; three symptom scales – fatigue, nausea/vomiting and pain; six single item scales – dyspnoea, insomnia, appetite loss, constipation, diarrhoea and financial impact; and the overall health/global QOL scale.

The EORTC QLQ-BN20, designed for use with patients undergoing chemotherapy or RT, includes 20 items assessing visual disorders, motor dysfunction, communication deficit, various disease symptoms (e.g. headaches and seizures), treatment toxicities (e.g. hair loss) and future uncertainty.

The items on both measures were scaled and scored using the recommended EORTC procedures (Fayers *et al*, 2001). Raw scores were transformed to a linear scale ranging from 0 to 100, with a higher score representing a higher level of functioning or higher level of symptoms. Provided at least half of the items in the scale were completed, the scale score was calculated using only those items for which values existed.

Patients were randomised following surgery and before the start of the RT. Radiotherapy started within 6 weeks after surgery. Valid HRQOL assessments were performed at baseline, before the start of therapy, not more than 1 month before randomisation and not more than 2 weeks after randomisation.

Health-related quality of life was a mandatory aspect of this clinical trial protocol. The protocol stipulated that a responsible nurse, clinician or data manager administered the questionnaire, requesting completion and its return to the EORTC Data Centre. The EORTC guidelines for administering questionnaires were provided, ensuring a standard approach and optimal compliance of HRQOL data collection by all personnel.

### Statistical analysis

For this analysis, only baseline HRQOL scores were used. To minimise the risk of false-positive results, we excluded *a priori* several HRQOL scales (seven from the QLQ-C30 and two from the brain module) from the analyses. The variables selected to be excluded were not expected to have any prognostic value or alternatively were known to be highly inter-correlated with other scales, thereby not contributing to the model. The final analysis included 17 HRQOL variables: eight (appetite loss, cognitive functioning, emotional functioning, fatigue, physical functioning, global health status, social functioning, insomnia) from the core questionnaire and nine (bladder control, communication deficit, drowsiness, future uncertainty, headaches, motor dysfunction, seizures, visual disorder, weakness of legs) from the brain module.

The Cox proportional hazards regression model (Cox, 1972) with overall survival measured from the time of randomisation as dependent outcome was used for both univariate and multivariate analyses. A Collett's Model Selection approach (Collett, 1994) was used with a level of significance of 0.15 for the univariate screening and stay and entry criterion of 0.05. The HRQOL scales were included as continuous factors. The model was controlled for the major established prognostic baseline clinical factors (Gorlia *et al*, in press): age (<50 *vs* ⩾50 years), performance status (0 *vs* 1 *vs* 2), extent of surgery (complete resection *vs* partial resection *vs* biopsy only), corticosteroids at entry (yes *vs* no), mini-mental state examination (<27 *vs* 27–30), *O*^6^-methylguanine-DNA methyltransferase (MGMT) promoter methylation status (yes *vs* no) as well as randomly assigned therapy. The MGMT status could only be assessed in 36% of the patients and was shown to be predictive of a favourable treatment effect in patients receiving TMZ chemotherapy (Hegi *et al*, 2005). The MGMT promoter methylation status was included in the analysis as a stratum with three levels (methylated, unmethylated or missing). The treatment assignment was also included as a stratum (because of the interaction between treatment and MGMT methylation). All other factors were included as covariates.

Validation of the final model was undertaken by use of several refined statistical techniques. The stability of the final model was investigated using a bootstrap re-sampling procedure as proposed by Sauerbrei and Schumacher (1992), applied in the context of HRQOL (Van Steen *et al*, 2002). This technique generates a number of samples (each of the same size as the original data set), by randomly selecting patients and replacing them before selecting the next patient (i.e. bootstrap resampling). The frequency of inclusion of the HRQOL scores in the Cox PH regression models, including all the selected clinical factors and treatment, fitted to each of these data sets using automatic forward stepwise selection (entry level of *α*=0.05), can be considered to be indicative for the importance of the HRQOL factors. This technique was applied to 1000 bootstrap samples.

Deviance residuals from the model with clinical factors were plotted *vs* the HRQOL scores to explore the relationship between each HRQOL score and the remaining part of the hazard not already explained by clinical factors. Finally, discrimination *C*-indexes and Nagelkerke's *R*^2^ coefficients were computed to quantify the predictive accuracy of a model. *C*-index is a measure of how well a model is able to rightly predict which patient among a randomly chosen pair of patients will survive longer. Nagelkerke's *R*^2^-coefficient is a generalisation of the *R*^2^-coefficient in linear regression, which measures the percentage of variation in the dependent variable accounted for by the predictor variables. It assesses the ability of the model to separate between all patient responses based on the predictor variables.

All data analyses were performed using Statistical Analysis Software (SAS) version 9.

## RESULTS

Between June 2000 and March 2002, 573 patients from 85 institutions in 15 countries were randomised to receive either RT alone (286 patients) or RT with TMZ (287 patients). Of these patients, 248 (RT) and 242 (RT plus TMZ) had baseline HRQOL measures completed. In total, 83 (RT) and 86 (RT plus TMZ) had an assessment of their methylation status of the MGMT promoter. The analysis was performed on 490 patients with baseline HRQOL measures that represent 85.5% of the original sample size.

### Clinical and quality-of-life results

The clinical results have been published (Hegi *et al*, 2005; Stupp *et al*, 2005; Mirimanoff *et al*, 2006). In brief, the study demonstrated that the addition of TMZ to RT for newly diagnosed glioblastoma resulted in a clinically meaningful and statistically significant survival benefit with minimal additional toxicity (unadjusted hazard ratio of 0.63 with a 95% confidence interval, 0.52–0.75; *P*<0.001 by the log-rank test). In addition, patients whose tumours had a methylated MGMT gene promoter benefited from TMZ, whereas those who did not have a methylated MGMT promoter derived no or only limited benefit from the addition of chemotherapy. Quality-of-life results have also been published (Taphoorn *et al*, 2005). Addition of TMZ during and after RT significantly improved survival without a negative effect on HRQOL. Baseline clinical characteristics for patients with a valid baseline HRQOL questionnaire are depicted in Table 1.

The distribution of the baseline clinical characteristics was very similar between patients with a valid baseline HRQOL questionnaire and those without. When comparing patients with and those without available MGMT assessment, imbalances were noticed for the clinical characteristics age, MMSE score, extent of surgery and tumour location. The percentages of patients under 50 years, with an MMSE above 27 or with a unifocal tumour location are higher in patients with a MGMT status. The subgroup of patients with MGMT promoter methylation status assessed was therefore not entirely representative of the whole trial population. A higher proportion of patients with resected tumours had the MGMT status successfully assessed due to the lack of a sufficient amount of tumour tissue in stereotactic biopsies.

### Prognostic factor analysis results

#### Univariate analysis

When controlled for the major prognostic clinical factors, the HRQOL scores that passed the 15% significance level were cognitive functioning, fatigue, global health status, social functioning, bladder control, headaches and motor dysfunction. Table 2 shows the results of this analysis.

### Multivariate analysis

The Cox multivariate model selected by Collett's Model Selection approach contained cognitive functioning, global health status and social functioning in addition to the selected clinical factors (Table 3). However, the sign of the coefficient related to social functioning was opposite to what was expected, that is, worse social functioning was related to better survival.

### Bootstrap re-sampling procedure

Table 4 presents the results of the bootstrap re-sampling procedure. The inclusion frequencies above 50% were related to cognitive functioning (83.0%), global health status (55.2%) and social functioning (88.9%). The frequencies of selection of each possible set of HRQOL scores were very low. The most frequently selected model (cognitive functioning, global health status, social functioning) was selected only 8.8% of the time. This indicates a high degree of model instability with no single model to be uniformly preferable over all others.

### Discrimination index *C* and Nagelkerke's *R*^2^ coefficient

Discrimination *C*-indexes and Nagelkerke's *R*^2^ coefficients were computed for the model with clinical prognostic factors only and for various models with added HRQOL scores. The *C*-index of the model with only clinical baseline characteristics is *C*=0.647 and the Nagelkerke's *R*^2^-coefficient is *R*^2^=0.133. When adding HRQOL scores, *C*=0.654 and *R*^2^=0.177 for the three selected HRQOL scores and *C*=0.647 and *R*^2^=0.189 for all HRQOL scores. For the model without clinical factors but with all HRQOL scores, *C*=0.604 and *R*^2^=0.104.

## DISCUSSION

Glioblastoma, like any other malignant brain tumour, have a considerable impact on the patient's HRQOL, and in spite of important progress due to the addition of chemotherapy to RT and surgery, as a rule most glioblastoma patients will eventually suffer from tumour relapse. With such a poor prognosis, evaluation of patients' HRQOL before and during treatments becomes important. In this study, we explored if HRQOL data provide reliable and useful prognostic information. Our study used 490 glioblastoma patients, controlled for major prognostic clinical factors, and attempted to overcome limitations of past studies by using a larger sample size of homogeneously treated patients and sophisticated statistical methodology.

Previous studies in brain tumour patients showed that HRQOL factors and/or cognitive functioning were statistically significant factors in prediction models for groups of patients, comparable to our classical analysis. For example, Sehlen *et al* (2003) examined HRQOL in 153 patients with either malignant astrocytoma or brain metastases. Using the Functional Assessment of Cancer Therapy General HRQOL measure (Cella *et al*, 1993), they found two variables, that is, ‘living with a spouse’ and the FACT-G total score to predict survival. Two other studies demonstrated objective cognitive functioning to have prognostic significance, both in newly diagnosed and in recurrent high-grade glioma (Meyers *et al*, 2000; Klein *et al*, 2003). Meyers *et al* (2000) examined HRQOL and cognitive functioning in 80 patients with recurrent malignant glioma or anaplastic astrocytoma, at baseline, before treatment in phase I and II trials. Health-related quality of life was undertaken with the FACT-BR module, along with other neuropsychological tests. Health-related quality-of-life scores did not predict survival, but cognitive functioning was a significant predictor of survival. It is difficult to compare these findings with our results, given the different measures that were used, along with their sample being relatively small. In addition, the Phase I/II setting of Meyers *et al* is likely to be considerably different (higher expectations and discounting toxicities) to that of a large phase III trial (Cheng *et al*, 2000). Klein *et al* (2003) explored cognitive functioning along with activities of daily living in 68 newly diagnosed high-grade glioma patients. Cognitive functioning had prognostic value, but only in a subsample of older patients. However, it is unclear to what extent studies on such small samples can be relied on for providing definitive conclusions. It is also difficult to make comparisons between our trial and Klein *et al*. due to the different HRQOL measures employed.

There are several issues questioning the validity and the reliability of the results obtained by classical techniques. Some of them are well known, such as the large number of HRQOL scales and the intercorrelation of these HRQOL scales. It makes the selection of a particular set of HRQOL scores quite difficult as various sets of HRQOL scores may predict equally survival when added to clinical factors. It may also lead to models difficult to interpret (with worst HRQOL status associated with longer survival) as some HRQOL factors may enter the model just as corrections for others. In addition, as HRQOL scores are analysed as continuous factors, the results could be influenced by a few ‘outliers’, that is, patients with some very bad HRQOL scores but who actually survived long or *vice versa*. Furthermore, the residuals plots also suggested a high variability in duration of survival among patients who have a same level of the HRQOL scores.

*C*-indexes and *R*^2^-coefficients are thought to better assess the potential benefit of using baseline HRQOL scores in addition to the clinical factors to predict survival in clinical practice and research. In our study, the calculated coefficients did not exhibit major improvement when adding selected or all HRQOL scores to clinical factors, suggesting that baseline HRQOL scores in the end add relatively little to known clinical factors to predict survival and cannot be used alone in outcome prediction.

Care needs to be taken when interpreting the results of our study, given our study had limitations, particularly as this was an exploratory analysis. Also, while 490 patients represent a considerable sample, other data sets are required to validate these findings.

In summary, while traditional methods of analysis suggest HRQOL data are prognostic, more detailed analysis revealed these findings may not be as reliable as expected. Further research should investigate the use of HRQOL with more sophisticated techniques to obtain reliable results.

The investigation of the prognostic value of HRQOL data is a challenging and ongoing research area. Further research could also investigate the prognostic value of changes from baseline in HRQOL rather than baseline values and should investigate why HRQOL parameters might be of value in one setting but not another. Furthermore, the reason for this association between HRQOL data and survival is unclear. Some hypotheses have been proposed to explain the mechanisms underlying the association. Patients' HRQOL scores might reflect an early perception of the severity of the disease in a more accurate way than conventional prognostic indices. In this case, patients who report worse HRQOL scores are the ones with a worse underlying disease. This hypothesis does not imply a true causative relationship between HRQOL parameters and survival. On the other hand, it is also possible that a better HRQOL score (which reflects a better physical and psychological state) could somehow have a positive effect on the disease process by, for example, slowing tumour progression. This causative explanation could be supported by some intervention studies which have shown that psychosocial support improved both psychological well-being and survival time. Coates *et al* (2000) assumed that, if the mechanism underlying the association between HRQOL and survival is causative, one should expect to see HRQOL parameters being prognostic of clinical outcomes, not only in patients with metastatic disease, but also at an earlier stage of the disease. Given this assumption, and the fact that their study did not find a correlation between HRQOL parameters and disease-free survival in their nonmetastatic breast cancer population, the authors argued in favour of the explanation that HRQOL scores reflect a more accurate perception of the severity of the underlying illness. The results of Efficace *et al* (2004a, 2004b) also seem to support this view. Hence, it would be possible to speculate that for early stage disease, clinical examinations (such as performance status or tumour staging) are more likely to supersede patients' self-reported HRQOL scores in predicting survival. However, more studies are required to definitively exclude any possible causative relationship with survival.

## Figures and Tables

**Table 1:** Baseline clinical characteristics of patients with HRQOL

**Baseline characteristics**			
	**Treatment**		
	**RT (*N*=248)**	**RT+TMZ (*N*=242)**	**Total (*N*=490)**
	***N* (%)**	***N* (%)**	***N* (%)**
*Age (years)*			
<50	72 (29.0)	75 (31.0)	147 (30.0)
⩾50	176 (71.0)	167 (69.0)	343 (70.0)
			
*Sex*			
Male	152 (61.3)	153 (63.2)	305 (62.2)
Female	96 (38.7)	89 (36.8)	185 (37.8)
			
*MMSE score*			
<27	78 (31.5)	68 (28.1)	146 (29.8)
27–30	167 (67.3)	168 (69.4)	335 (68.4)
Missing	3 (1.2)	6 (2.5)	9 (1.8)
			
*PFS (WHO)*			
0	95 (38.3)	96 (39.7)	191 (39.0)
1	120 (48.4)	113 (46.7)	233 (47.6)
2	33 (13.3)	33 (13.6)	66 (13.5)
			
*Corticosteroids at entry*			
No	63 (25.4)	78 (32.2)	141 (28.8)
Yes	185 (74.6)	164 (67.8)	349 (71.2)
			
*Extent of surgery*			
Biopsy only	39 (15.7)	38 (15.7)	77 (15.7)
Partial resection	116 (46.8)	107 (44.2)	223 (45.5)
Total resection	93 (37.5)	97 (40.1)	190 (38.8)
			
*Tumour location*			
Unifocal	206 (83.1)	198 (81.8)	404 (82.4)
Multifocal+other	42 (16.9)	44 (18.2)	86 (17.6)
			
*MGMT*			
Methylated	38 (15.3)	40 (16.5)	78 (15.9)
Unmethylated	48 (19.4)	49 (20.2)	97 (19.8)
Missing	162 (65.3)	153 (63.2)	315 (64.3)

**Table 2:** Univariate prognostic factor analyses for survival (controlled for clinical factors)

**HRQOL scores^a^**	**Hazard ratio (HR)**	**95% confidence interval (CI)**		***P***-**value**
*EORTC QLQ-C30*				
Appetite loss	1.017	0.967	1.069	0.5088
Cognitive functioning	0.919	0.887	0.953	<0.0001
Emotional functioning	0.983	0.943	1.025	0.4162
Fatigue	1.063	1.022	1.106	0.0025
Physical functioning	0.927	0.890	0.967	0.0004
Global health status	0.939	0.901	0.977	0.0022
Social functioning	1.012	0.981	1.044	0.4504
Insomnia	0.994	0.961	1.029	0.7308
				
*EORTC QLQ-BN20*				
Bladder control	1.066	1.019	1.115	0.0055
Communication deficit	1.088	1.046	1.132	<0.0001
Drowsiness	1.021	0.982	1.061	0.2967
Future uncertainty	1.019	0.983	1.055	0.3051
Headaches	1.024	0.984	1.065	0.2373
Motor dysfunction	1.092	1.048	1.139	<0.0001
Seizures	1.079	1.020	1.140	0.0079
Visual disorder	1.028	0.966	1.093	0.3857
Weakness of legs	1.044	1.005	1.084	0.0276

a For every 10 point shift in the scale ranging between 0 and 100.

**Table 3:** Multivariate model predicting survival for patients with HRQOL

**Variables**	**Hazard ratio (HR)**	**95% confidence interval (CI)**		***P***-**value**
*Clinical variables*				
Age (<50 *vs* ⩾50 years)	1.274	1.008	1.611	0.0430
Biopsy *vs* partial resection	0.830	0.614	1.121	0.2246
Biopsy *vs* total resection	0.618	0.448	0.853	0.0034
Performance status (0 *vs* 1)	1.069	0.844	1.354	0.5803
Performance status (0 *vs* 2)	1.170	0.825	1.659	0.3780
MMSE (<27 *vs* 27–30)	0.679	0.536	0.861	0.0014
Corticosteroids at entry (no *vs* yes)	1.583	1.242	2.016	0.0002
				
*HRQOL scores* a				
Cognitive functioning	0.918	0.878	0.959	0.0001
Global health status	0.929	0.882	0.979	0.0055
Social functioning	1.090	1.046	1.137	<0.0001

a For every 10 point shift in the scale ranging between 0 and 100.

**Table 4:** Inclusion frequencies in the bootstrap re-sampling procedure

**Variable**	**Inclusion (%)**	**Pos. incl. (%)**	**Neg. incl. (%)**
Age (<50 *vs* ⩾50)	100.0	96.9	3.1
Partial resection *vs* biopsy	100.0	14.0	86.0
Total resection *vs* biopsy	100.0	1.3	98.7
Performance status (1 *vs* 0)	100.0	75.9	24.1
Performance status (2 *vs* 0)	100.0	67.9	32.1
MMSE (<27 *vs* 27–30)	100.0	0.3	99.7
Corticosteroids at entry (yes *vs* no)	100.0	99.9	0.1
Appetite loss	8.6	5.9	2.7
Cognitive functioning	83.0	0.0	83.0
Emotional functioning	7.5	6.6	0.9
Fatigue	8.6	7.7	0.9
Physical functioning	6.6	2.8	3.8
Global health status /QoL	55.2	0.0	55.2
Social functioning	88.9	88.9	0.0
Insomnia	20.5	0.1	20.4
Bladder control	24.2	24.2	0.0
Communication deficit	11.9	0.9	11.0
Drowsiness	5.2	2.3	2.9
Future uncertainty	27.4	27.3	0.1
Headaches	8.9	7.9	1.0
Motor dysfunction	20.0	20.0	0.0
Seizures	13.4	13.1	0.3
Visual disorder	9.4	5.8	3.6
Weakness legs	20.6	1.0	19.6

Positive inclusion is any inclusion of the factor in a model with a positive coefficient.

Negative inclusion is any inclusion of the factor in a model with a negative coefficient.
